# In-vivo and in-silico studies to identify toxicity mechanisms of permethrin with the toxicity-reducing role of ginger

**DOI:** 10.1007/s11356-023-31729-5

**Published:** 2024-01-08

**Authors:** Damla Himtaş, Emine Yalçin, Kültiğin Çavuşoğlu, Ali Acar

**Affiliations:** 1 Department of Biology, Institute of Natural Sciences, University of Giresun, 28200 Giresun, Turkey; 2 Department of Biology, Faculty of Science and Art, University of Giresun, 28200 Giresun, Turkey; 3 Department of Medical Services and Techniques, Vocational School of Health Services, University of Giresun, 28200 Giresun, Turkey

**Keywords:** *Allium cepa*, Genotoxicity, Molecular docking, Oxidative damage, Permethrin, *Zingiber officinale*

## Abstract

In this study, the toxic effects of permethrin on *Allium cepa* L. and the protective role of *Zingiber officinale* rhizome extract (Zoex) were investigated. In this context, 6 different groups were formed. While the control group was treated with tap water, the groups II and III were treated with 10 µg/mL and 20 µg/mL Zoex, respectively, and the group IV was treated with 100 µg/L permethrin. The protective effect of Zoex against permethrin toxicity was studied as a function of dose, and groups V and VI formed for this purpose were treated with 10 µg/mL Zoex + 100 µg/L permethrin and 20 µg/mL Zoex + 100 µg/L permethrin, respectively. After 72 h of germination, cytogenetic, biochemical, physiological, and anatomical changes in meristematic cells of *A. cepa* were studied. As a result, permethrin application decreased the mitotic index (MI) and increased the frequency of micronuclei (MN), and chromosomal abnormalities. The increase in malondialdehyde (MDA), superoxide dismutase (SOD), and catalase (CAT) and the decrease in glutathione (GSH) indicate that permethrin causes oxidative damage. Compared to the control group, a 68.5% decrease in root elongation (*p* < 0.05) and an 81.8% decrease (*p* < 0.05) in weight gain were observed in the permethrin-treated group. It was found that the application of Zoex together with permethrin resulted in regression of all detected abnormalities, reduction in the incidence of anatomical damage, MN and chromosomal aberrations, and improvement in MI rates. The most significant improvement was observed in group VI treated with 20 µg/mL Zoex, and Zoex was also found to provide dose-dependent protection. The toxicity mechanism of permethrin was also elucidated by molecular docking and spectral studies. From the data obtained during the study, it was found that permethrin has toxic effects on *A*. *cepa*, a non-target organism, while Zoex plays a protective role by reducing these effects.

## Introduction

In agricultural applications, many techniques are used in order to increase the quality of the products and the yield obtained from the unit area. Chemical control against microorganisms, insects, various diseases, and weeds is one of the important methods. Among all control methods, chemical control accounts for a high share of 95% and remains valid today. Pesticides are widely used in chemical control, and thus, the damage that may occur in the yield and quality of the products can be prevented (Dubus et al. 2000; Tiryaki et al. 2010). The use of pesticides, which show chemical changes according to the target organisms, has increased day by day, and intense pesticide pollution has occurred with the production of synthetic pesticides. Many pesticides exhibit cumulative properties by being transported between ecosystems and can be transported between organisms through food chains by accumulating in many organisms (Dubus et al. 2000; Junquera 2021). Pyrethroids constitute a large class of pesticides and are a group of natural insecticides obtained by extraction of the dried flowers of the chrysanthemum plant. However, natural pyrethroids have been replaced by synthetic pyrethroids over time due to their rapid degradation in the environment. Permethrin belongs to the synthetic pyrethroid chemical class and is classified as a type I pyrethroid. Permethrin is used for protection from fleas, microorganisms, and parasites in sectors such as agriculture and livestock. Permethrin, which is also used in industrial areas, is used to protect clothes from insects and pests, especially in the ready-made clothing industry. Permethrin has also found widespread use in the treatment of parasites such as head lice, fleas, and especially scabies, and for this purpose, it is also found in scabies creams, scabies soaps, and in the content of all kinds of care and treatment products related to scabies (Junquera 2021). Because of its widespread use, permethrin contaminates the environment in several ways and has toxic effects on non-target organisms. Permethrin exposure disrupts the balance of the antioxidant system in non-target organisms and causes oxidative damage to DNA, lipids, and proteins (Weidinger and Kozlov 2015). Sun et al. (2022) reported that long-term and low-dose exposure to permethrin caused liver and kidney damages in non-target rats. Davoodi et al. (2012) found that high mortality rates were observed in juvenile *Cyprinus carpio* treated with permethrin in the dose range of 10–125 mg/L. Studies investigating the effects of permethrin on non-target plants are still insufficient. In this study, the toxic effects of permethrin on root tip cells of *Allium cepa*, one of the non-target organisms, were investigated using a multidisciplinary approach.

Natural products with antioxidant properties regulate the antioxidant balance, which is deteriorated due to the chemical load in organisms. In this context, the toxicity-reducing effect of *Zingiber officinale* rhizomes (ginger), which has antioxidant properties, was also investigated in this study. Rhizome of *Z. officinale* has been used since ancient times for colds, coughs, various infections, fever, asthma, bronchitis, anorexia, arthritis, rheumatism, edema, pain, cramps, nausea, vomiting, flatulence, gastritis, peptic ulcer, intestinal parasites, hemorrhoids, constipation, hypertension, dementia, dysmenorrhea, stroke, diabetes, and various nervous system disorders. The powdered rhizome of *Z. officinale* contains 3–6% lipid, 9% protein, 60–70% carbohydrate, 3–8% crude fiber, approximately 8% ash, 9–12% water, and 2–3% essential oil (Policegoudra et al. 2010; Banerjee et al. 2011). Rhizomes contain the minerals such as calcium, phosphorus, and iron and include vitamins like thiamine, vitamin C, niacin, and riboflavin (Govindarajan 1982). The activity of *Z. officinale* is closely related to its active ingredients, and the gingerol, zingerone, and shogaol it contains are responsible for most of its biological effects (Kim et al. 2017). Due to the cumulative effect of all these phytochemicals, *Z. officinale* has potent antioxidant activity. *Z. officinale* exhibits antioxidant activity through various mechanisms due to the active compounds they contain and provide protection against numerous diseases caused by oxidative stress. Increasing the expression of antioxidant enzymes, preventing the formation of free radicals, preventing lipid peroxidation, and stimulating glutathione synthesis are some of these mechanisms. *Z. officinale* and its bioactive compounds exert their antioxidant effects through the nuclear factor erythroid 2-related factor 2 (Nrf2) pathway (Mao et al. 2019).

In this study, permethrin toxicity and protective properties of *Z. officinale* rhizomes extract (Zoex) against this toxicity were investigated by a bioindicator test. The *Allium* test is a method used as a bioindicator in which the effects of environmental pollution and toxic agents are examined and shows a high correlation with toxicity tests performed in mammals (Kutluer et al. 2019; Yalçin and Çavuşoğlu 2022a). From this point of view, in this study, the effects of permethrin and Zoex on *A. cepa* were investigated in terms of physiological, cytogenetic, biochemical, and anatomical aspects. The toxicity of permethrin studied with different parameters was supported by in silico and spectral methods. The interactions of permethrin with tubulin, histone, and DNA molecules were studied by in silico molecular docking. Tubulin proteins are located in the structure of the spindle responsible for pulling chromosomes to the poles during cell division. Structural disruptions that can occur in the spindle can lead to aneugenic effects and cell cycle delays. The interaction between histone and DNA is very important for maintaining genome integrity. Compounds that bind to histones or DNA pose a significant risk for disrupting this integrity, and the genotoxic potential of permethrin was determined by studying permethrin-histone and permethrin-DNA interactions. In this study, permethrin toxicity and the effects of Zoex application, which will be a solution to reducing this toxicity, were also investigated. The protective effect of Zoex against permethrin toxicity is also associated with the major components such as gingerol, zingerone, and shogaol.

## Material and methods

### *Zingiber* officinale extraction

The rhizomes of *Z. officinale* were dried, and after grinding, 0.2 g of the sample was extracted in 10 mL of methanol for 24 h at room temperature. After incubation, the extract was filtered to remove all remaining solids. The filtrate was then centrifuged at 10,000 rpm for 10 min, the liquid phase was evaporated, and the pellet was used as *Z. officinale* extract (Akgeyik et al. 2023).

### Experimental groups

**Fig. 1:**
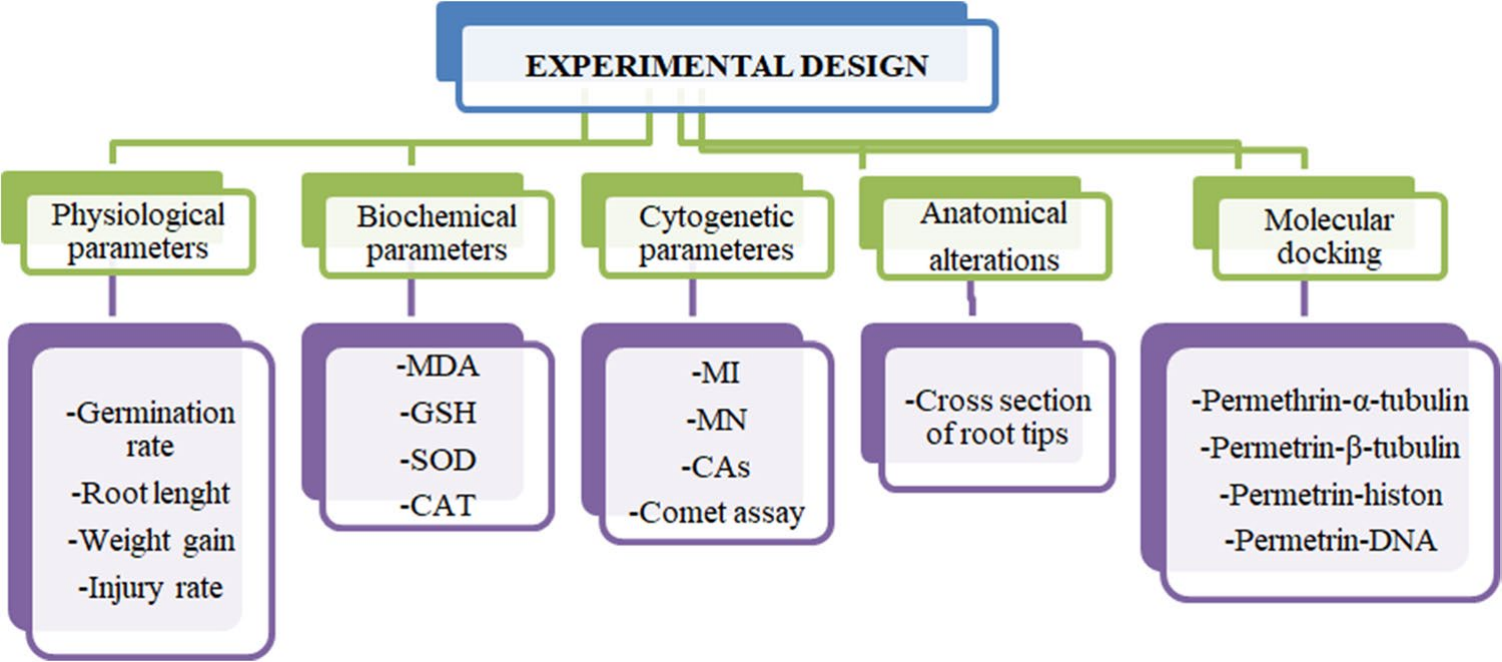
All parameters investigated in experimental stages

The toxicity of permethrin and the protective role of Zoex were investigated using the *Allium* test. The bulbs of *A. cepa* were obtained from a commercial market. To determine the permethrin toxicity and the protective role of Zoex, 6 different groups were formed. The control group (group I) was treated with tap water. The groups II and III were treated with 10 µg/mL Zoex and 20 µg/mL Zoex, respectively, and it was tested whether Zoex alone had a toxic effect in these groups. Bulbs in the group IV were treated with 100 µg/L permethrin. The protective effect of Zoex against permethrin toxicity was studied as a function of dose, and groups V and VI formed for this purpose were germinated with 10 µg/mL Zoex + 100 µg/L permethrin and 20 µg/mL Zoex + 100 µg/L permethrin, respectively. Germination occurred at 24 °C for 72 h (Akgündüz et al. 2020). Several parameters were studied to determine the permethrin toxicity and the protective effect of Zoex. The parameters studied are shown in Fig. 1.

### Germination related parameters

The effects of Zoex and permethrin on germination were determined by weight gain, root length, germination percentage (GP), and relative injury rate (RIR) parameters. Root growth was determined by determining radicle length and weight gain by measuring the weight of each bulb before and after application. GP was calculated using Eq. (1) (Yalçin and Çavuşoğlu 2022b). For the germination test, 50 bulbs were tested, and for the root length and weight gain analysis, 10 bulbs were tested. The RIR was calculated using Eq. (2).

1 $$\text{GP}\;\left(\%\right):\left[\mathrm{Germinated}\;\mathrm{bulb}\;\mathrm{number}/\mathrm{Total}\;\mathrm{bulb}\;\mathrm{number}\right]\times100$$

2 $$\text{RIR}=\left[\%\mathrm{GP}\;\mathrm{of}\;\mathrm{control}-\%\mathrm G\mathrm P\;\mathrm o\mathrm f\;\mathrm t\mathrm r\mathrm e\mathrm a\mathrm t\mathrm m\mathrm e\mathrm n\mathrm t\;\mathrm g\mathrm r\mathrm o\mathrm u\mathrm p\right]/\left[\%\mathrm{GP}\;\mathrm{of}\;\mathrm{control}\right]$$

### Cytogenetic parameters

To detect the chromosomal abnormalities and micronucleus frequency and to evaluate MI, root tip samples were collected from each group and subjected to ethanol series and hydrolysis. Samples stained overnight with acetocarmine (5%) were examined by two different observers. A total of 1000 cells were analyzed for MN and chromosomal aberration analysis. MI was calculated using Eq. (3) and determined by analyzing 10,000 cells from each group (Tütüncü et al. 2019).

3 $$\text{MI}\;\left(\%\right)=\left[\mathrm{number}\;\mathrm{of}\;\mathrm{dividing}\;\mathrm{cells}/\mathrm{total}\;\mathrm{number}\;\mathrm{of}\;\mathrm{cells}\right]\times100$$

### Comet test

Comet analysis was applied according to the protocol suggested by Chakraborty et al. (2009). For nucleus isolation, root tip samples from each group were gently crushed in Tris buffer and placed in 1% NMPA solution. Forty microliters of suspension and 40 µL of 1% LMPA were mixed gently, and a coverslip was placed on the mixture. After solidification, the coverslip was removed and a layer of 80 μL of 0.5% LMPA was created on the surface. Slides were transferred to a gel electrophoresis tank containing Na2EDTA and NaOH (pH > 13) and electrophoresis was performed for 20 min at 4 °C and 0.7 V/cm (20 V and 300 mA). At the end of electrophoresis, the slides were rinsed with Tris buffer and stained with ethidium bromide (20 μg/mL) for 5 min. Comet scores (tail length) were analyzed with the help of Comet Assay Software (CASP-version 1.2.3b) (Końca et al. 2003). A total of 1000 cells per group, 100 in each bulb, were analyzed for DNA damage. Comet analyses were repeated twice with CASP on the slides prepared for each group. Cells were analyzed for five categories, from zero to four, according to varying tail DNA lengths as stated by Collins (2004). Total DNA damage per group was calculated using Eq. (4).

4 $$\mathrm{Arbitrary}\;\mathrm{unit}=\sum\nolimits_{i=0}^4Ni\;x\;i$$

Ni, the number of cells in *i* degree; *i*, degree of damage (0, 1, 2, 3, 4).

### In silico study on permethrin interactions with cellular molecules

In order to elucidate the toxicity mechanism of permethrin, its interaction with cellular macromolecules was also investigated in silico. Regarding the selection of cellular macromolecules, tubulin and histone proteins were chosen due to their essential roles in cellular processes. Tubulins are crucial components of microtubules, vital for cell division and structure, while histones play a central role in DNA packaging and gene regulation (Çakir et al. 2023). By studying how permethrin interacts with these macromolecules, insights into its potential effects on cellular processes and genotoxic mechanisms were gained. Permethrin-tubulin proteins were examined to evaluate the spindle fiber damage causing an anogenic effect, and permethrin-histone and permethrin-DNA interactions were examined by molecular docking to evaluate the clastogenic effect. The structures of alpha-1B chain and tubulin beta chain (6RZB) (Lacey et al. 2019), histone H2A.6 and histone H2B.1 (7BP2) (Luo et al. 2020), B-DNA dodecamer (PDB ID: 1bna) (Drew et al. 1981), DNA (PDB ID: 1cp8) (Katahira et al. 1998), and B-DNA dodecamer d (PDB ID: 195d) (Balendiran et al. 1995) were obtained from the protein data bank. The 3D structure of permethrin (PubChem CID: 40326) was retrieved from the PubChem. Energy minimization of proteins was applied with Gromos 43B1 using Swiss-PdbViewer (Guex and Peitsch 2005) (v.4.1.0) software whereas energy minimization of permethrin was accomplished with the UFF-force field employing Open Babel v.2.4.0 software (O’Boyle et al. 2011). Molecular docking was performed using Autodock 4.2.6 software (Morris et al. 2009). The scoring functions and parameters used in the molecular docking were selected to evaluate the binding affinities of permethrin with the target macromolecules. The free energy of binding (in kcal/mol) and inhibition constants (Ki) were assessed, providing quantitative measures of the strength of ligand-protein interactions.

### Spectral analysis of permethrin-DNA interaction

To confirm the DNA-permethrin interaction, which was demonstrated by molecular docking, the changes in the UV spectrum of DNA isolated from *Allium* were investigated. DNA was isolated from root tip cells of *A. cepa* according to the method developed by Sharma et al. (2002). DNA-permethrin interactions were evaluated by measuring the absorbances of the DNA solution at different wavelengths in the presence and absence of permethrin (DNA/permethrin, 1:0, 1:1, 1:2, 1:4) by using Mapada UV-6100PCS double beam spectrophotometers.

### Disruptions in antioxidant-oxidant balance

The biochemical effects of permethrin and Zoex application were determined by investigating the changes in antioxidant and oxidant balance. The levels of superoxide dismutase and catalase (CAT), glutathione (GSH), and the oxidant molecule malondialdehyde (MDA) were measured. Root tissues were extracted before biochemical analysis. Root tip samples (0.5 g) were extracted in phosphate buffer and the supernatant obtained after centrifugation was used for analysis (Yalçın et al. 2019). SOD activity was determined according to the method proposed by Aydin et al. (2022). A mixture of sodium phosphate buffer (1.5 mL), nitroblue tetrazolium chloride (0.3 mL), methionine (0.3 mL), riboflavin (0.3 mL), EDTA-Na_2_ (0.3 mL), insoluble polyvinylpyrrolidone (0.01 mL), extract (0.01 mL), and deionized water (0.28 mL) was held under a fluorescent lamp (15 W) for 10 min. At the end of the time, the reaction was terminated by keeping it in the dark and the absorbance of the solution was read at 560 nm and activity was expressed as U/mg FW. For CAT activity measurements, 2.8 mL of reaction mixture was prepared monosodium phosphate buffer (1.5 mL), distilled water (1.0 mL), and hydrogen peroxide (0.3 mL). The reaction was initiated by adding 0.2 mL of the extract. The activity of CAT was measured by monitoring the decrease in absorbance at 240 nm, and the activity of CAT is expressed as U/mg FW (Demirtaş et al. 2020). In order to determine the change in antioxidant/oxidant enzyme levels, MDA and GSH levels were measured as well as antioxidant enzymes. A 5% thiobarbituric acid (1:1) was added to the root homogenate and incubated at 96 °C for 25 min for MDA analysis. After incubation at high temperatures, the mixture was centrifuged at 10,000 rpm and absorbance was measured at 532 nm. MDA concentration was determined as µM/g FW (Macar et al. 2020). GSH analysis was performed using the Kurt et al. (2023) protocol. The measurement of each parameter was performed in triplicate.

### Recovery effects of Zoex

The recovery effects of Zoex were determined by using the data of the permethrin + Zoex applied groups, the data of the 100 µg/L permethrin application group, and the data of the control group (Eq. 5).

5 $$\mathrm{Recovery}\;\mathrm{effect}\;\left(\%\right)=\left[\left({\text{D}}_1-{\text{D}}_2\right)/\left({\text{D}}_3-{\text{D}}_2\right)\right]\times100$$

*D*_1_, data of permethrin + Zoex-treated group; *D*_2_, data of 100 µg/L permethrin-treated group; *D*_3_, data of the control group.

### Anatomical alterations

To determine the anatomical changes in the root tips, cross-sections were taken from the roots of each group. Sections were stained with methylene blue (5%) for 4 h. The root sections of each group were examined with a research microscope and the frequency of abnormalities was determined (Çavuşoğlu and Yalçın 2023).

### Statistical analysis

The “IBM SPSS Statistics 22” package program was preferred for statistical analysis of the data. Statistical significance between all data given as mean ± SD was determined by the one-way ANOVA and the Duncan test and was considered statistically significant when *p* < 0.05.

## Results and discussion

### Germination-related parameters

The effects of permethrin and Zoex applications on germination parameters in *A. cepa* are given in Table 1. There were no statistical differences in terms of GP, root length, and weight gain in the control, groups II and III treated with Zoex alone. GP was found to be 65% in the permethrin-treated group (group IV), representing a 1.53-fold decrease compared to the control. Similar reductions were observed in the root length and weight gain, with a 68.5% decrease in root elongation and an 81.8% decrease in weight gain in the permethrin-treated group compared to the control group. Among all treatment groups, the highest damage rate, at 0.53, was observed in the permethrin-applied group. Abnormalities in germination-related parameters can be explained by the cellular toxicity induced by permethrin. The physiological responses of plants to pesticides are closely related to photosynthesis or oxidative stress. Pesticides can have a direct phytotoxic effect by disrupting the photosystem II, chlorophyll, or chloroplast biosynthesis. They also induce the production of reactive oxygen species and can have an indirect effect by causing damage to cellular components through oxidative stress. Both the direct and indirect effects can lead to disruption of physiological responses and growth arrest in plants. Pyrethroid insecticides, including permethrin, cause inhibition of the development of root and stem shoots in plants, and changes in photosynthetic pigment levels, causing abnormalities in non-target organism plants (Tang et al. 2018). In cells exposed to permethrin, disruption of the antioxidant system balance and oxidative damage to macromolecules such as DNA, lipid, and protein occurs (Weidinger and Kozlov 2015). However, permethrin also causes inhibition of complexes in the electron transfer chain system, and this effect causes disruption in photosynthetic processes and delays the germination process (Falcioni et al. 2010). Due to the adverse effects of permethrin, the germination and development of *A. cepa* are negatively impacted. Çavuşoğlu et al. (2012) reported that a pyrethroid insecticide cypermethrin significantly reduced the photosynthetic pigment levels in *A. cepa*, and this decrease was associated with the stress occurring in the cell and the inhibition of biosynthesis mechanisms. Borowik et al. (2023) emphasized that permethrin application in *Zea mays* caused a 37.9% decrease in the yield of the aerial parts and a 33.9% decrease in the roots. There was a dose-related amelioration in germination parameters in groups treated with permethrin + Zoex. The root length increased by 52.1% and weight gain increased by 76% in group VI applied with 20 µg/mL Zoex + permethrin compared to the group in which only permethrin was applied. While the damage rate calculated based on the germination rates was 0.53 in the permethrin-applied group, it decreased to 0.29 in the group treated with 10 µg/mL Zoex + permethrin and to 0.21 in the group VI treated with 20 µg/mL Zoex + permethrin. These results show that the toxic effects of permethrin on germination regress in the presence of Zoex, and Zoex has a protective effect. The protective feature of Zoex can be explained by neutralizing the oxidative stress induced by permethrin. It is known that Zoex contains many components such as phenolic compounds, shogaol, paradol, gingerol, zingiberol, zingiberen, bisapolene, and vitamins A, C, and E (Al-Nahain et al. 2014). These active compounds protect cells against the actions of toxic agents. The protective effect of Zoex is related to the biological activities of the active phytochemicals in its composition. As a result, permethrin caused a regression in the parameters related to germination in *A. cepa*, while the Zoex application provided a dose-dependent improvement.

**Table 1:** Effects of permethrin and Zoex on germination parameters

Groups	GP (%)	Root length (cm)	Initial weight (g)	Final weight (g)	Weight gain (g)	Relative injury rate
I	100	7.30 ± 1.50^a^	7.97 ± 1.92	14.37 ± 2.61	6.40^a^	ND
II	100	7.50 ± 1.70^a^	7.83 ± 1.85	14.45 ± 2.63	6.62^a^	ND
III	100	7.70 ± 1.80^a^	7.90 ± 1.88	14.75 ± 2.70	6.85^a^	ND
IV	65	2.30 ± 0.80^d^	7.88 ± 1.86	9.04 ± 1.88	1.16^d^	0.53
V	71	3.60 ± 1.00^c^	7.80 ± 1.83	10.80 ± 1.92	3.00^c^	0.29
VI	79	4.80 ± 1.30^b^	7.86 ± 1.82	12.71 ± 1.96	4.85^b^	0.21

Values shown with different letters in the same column are statistically significant

*ND* not determined

### Antioxidant/oxidant dynamic

**Fig. 2:**
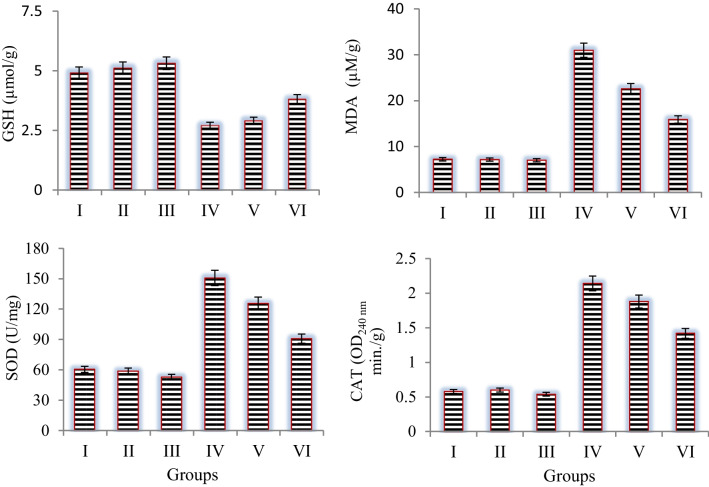
Effects of permethrin and Zoex on antioxidant/oxidant dynamics

To study the effect of permethrin and Zoex on the balance between antioxidants and oxidants, the levels of CAT, SOD, GSH, and MDA were measured (Fig. 2). A significant increase in antioxidant enzyme activities was observed in the group IV in which permethrin was administered. The increase in enzyme activities due to permethrin application indicates that the cell is protected against oxidative stress. Oxidative stress leads to oxidation of lipids, proteins, and nucleic acids and inhibition of enzymes. Antioxidant enzymes are stimulated in the cell against this oxidative stress-induced damage (Ahmad et al. 2010). In this study, the increase in SOD and CAT enzymes in the permethrin-treated group shows that permethrin application causes oxidative stress. Similarly, Çavuşoğlu et al. (2012) reported that low doses of cypermethrin, a pyrethroid insecticide, trigger CAT activity. Abnormalities in GSH and MDA levels also indicate oxidative stress. While GSH levels decreased by 44.8% in the permethrin-treated group, MDA levels increased by 76% compared to control. This result indicates that permethrin induces oxidative stress and, in particular, causes lipid peroxidation. MDA is the by-product of peroxidation of unsaturated fatty acids. The fact that permethrin application causes an increase in MDA levels in *A. cepa* indicates the development of oxidative stress and lipid peroxidation. Most higher plant tissues, cells, and organelles contain GSH. Among many other antioxidant properties, GSH directly interacts with and scavenges free radicals (Hausladen and Alscher 1993; Kerksick and Willoughby 2005). It is suggested that the decrease in GSH levels in the permethrin-treated group is due to the oxidation of GSH during free radical scavenging. Similarly, Çavuşoğlu et al. (2014) found that lambda-cyhalothrin, a pesticide like permethrin, caused an increase in MDA levels in *Allium* root tip cells. Gabbianelli et al. (2013) reported that administration of 150 mg/kg permethrin increased lipid peroxidation and decreased GSH and CAT activity in rats. Co-administration of Zoex and permethrin improved the antioxidant-oxidant balance. In the group receiving 20 µg/mL Zoex + permethrin, a 2.2-fold decrease in MDA levels and a 1.3-fold increase in GSH levels were observed compared to the group receiving permethrin alone. This shows that the antioxidant power in the cell increases. SOD and CAT activities, which increased significantly in the permethrin-treated group, also began to decrease. In the group receiving 20 µg/mL Zoex + permethrin, SOD and CAT activity decreased by 1.5–1.6 times compared to the group receiving permethrin alone. The antioxidant balance-improving effect of Zoex is due to the active constituents of the content, especially gingerol and shogaol. Gingerol and shogaol are potent antioxidant compounds that exhibit antioxidant activity in several ways. Their main antioxidant effect is inhibition of the production of ROS. Gingerol and shogaol inhibit the formation of hydroxyl radicals produced through the Fenton reaction (Kuhad et al. 2006; Dugasani et al. 2020). Moreover, by increasing the expression of genes involved in glutathione synthesis, such as glutamate cysteine ligase and glutamate cysteine ligase, 6-shogaol also increases intracellular GSH levels and provides significant protection (Mao et al. 2019). Co-administration of Zoex and permethrin improved the antioxidant-oxidant balance. In the group receiving 20 µg/mL Zoex + permethrin, a 2.2-fold decrease in MDA levels, a 1.5- to 1.6-fold decrease in SOD and CAT activities and a 1.3-fold increase in GSH levels were observed. The antioxidant balance-improving effect of Zoex is due to the active constituents of the content, especially gingerol and shogaol. Gingerol and shogaol are potent antioxidant compounds that exhibit antioxidant activity in several ways. Their main antioxidant effect is inhibition of the production of ROS. Gingerol and shogaol inhibit the formation of hydroxyl radicals produced through the Fenton reaction (Kuhad et al. 2006; Dugasani et al. 2020). Moreover, by increasing the expression of genes involved in glutathione synthesis, such as glutamate cysteine ligase and glutamate cysteine ligase, 6-shogaol also increases intracellular GSH levels and provides significant protection (Mao et al. 2019). As a result, permethrin caused deterioration in the antioxidant/oxidant balance in *A. cepa*, while Zoex application exhibited protective effects by increasing the GSH level and decreasing the MDA level.

### Cytotoxic effects

**Fig. 3:**
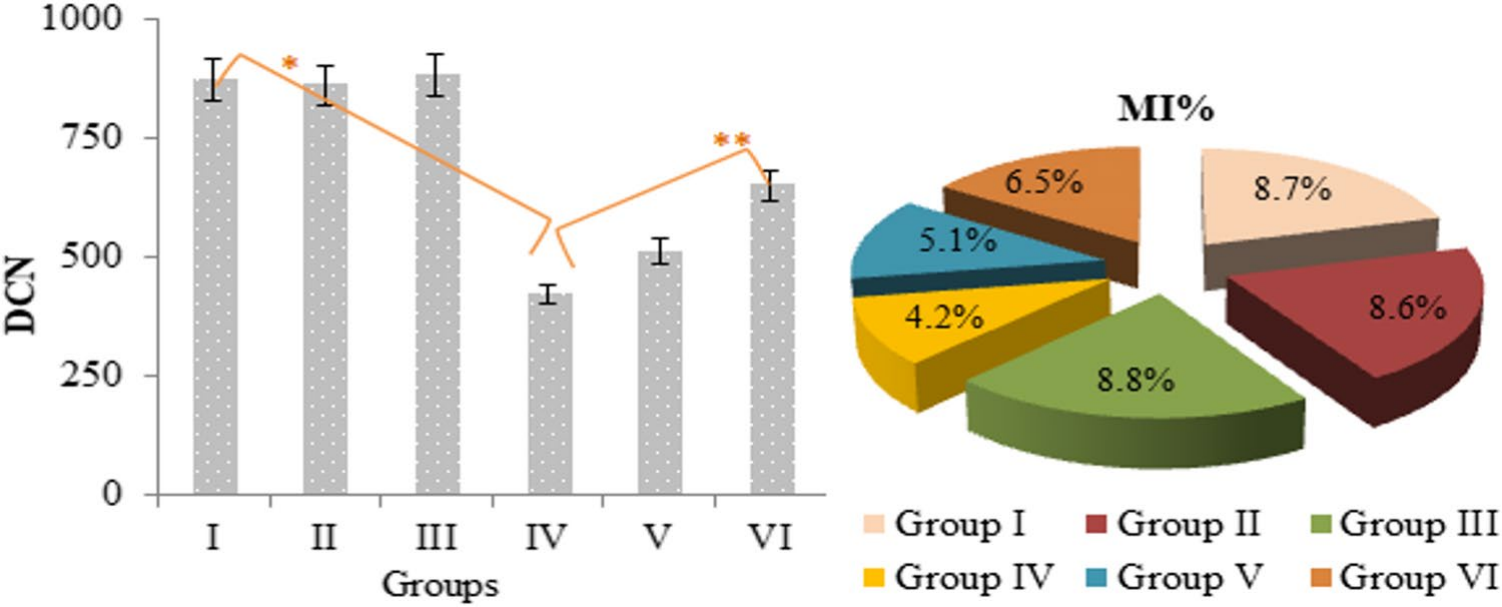
Effects of permethrin and Zoex on dividing cell number and MI. Asterisk (*) indicates statistical significance between control and group IV, and ** indicates statistical significance between groups IV and VI

The cytogenetic effects of permethrin and Zoex were investigated with MI rate and the frequencies of MN and chromosomal aberrations. The number of dividing cells within 10,000 cells counted in the control and Zoex-treated groups was found to be in the range of 860.48–880.90. In the permethrin-treated group, dividing cells decreased by 51.6% to 420.66 (Fig. 3). MI is a reliable indicator of cytotoxicity in living cells. MI rate decreases of less than 50% indicate a sublethal effect, while reductions above 50% indicate a lethal effect. According to this distinction, it has been observed that permethrin may exhibit a lethal effect in root tip cells of *A. cepa*. Cytotoxic agents show their reducing effect on MI rates by inhibiting microtubule formation. Chromosomal aberrations such as multipolar anaphase, c-mitosis, and sticky chromosome are also associated with inhibition of microtubule and spindle fiber formation. In the permethrin-treated group, a high frequency of MN formation was also observed, along with a decrease in the MI rate. The formation of MN was detected in a total of 72.84 cells in the group treated with permethrin (Table 2). The high frequency of MN, detected in the permethrin group, also supports the decrease in the MI rate. Spindle abnormalities, which lead to a decrease in the MI rate, can also trigger the formation of MN (Fenech and Neville 1992). Delic (1998) found that the administration of permethrin at concentrations of 1–12 mg/mL significantly reduced the MI rate in cell cultures and showed cytostatic effects through cell cycle regression. Roma et al. (2012) reported that the permethrin application resulted in damage to genetic material and the formation of MN was observed in the first cell cycle after 24 h of application. Abnormalities observed in MI and MN rates were found to regress in the groups treated with permethrin + Zoex. The administration of 20 µg/mL Zoex with permethrin caused a 38.16% increase in the MI rate and a 52.38% decrease in MN frequency compared to group IV. This result shows the protective effects of Zoex against permethrin-induced cytotoxicity. Permethrin generally causes oxidative stress in cells and oxidation in macromolecules. One of the main target molecules of permethrin-induced oxidative damage is proteins. It is known that the formation of carbonyl proteins increases as a result of oxidation induced by permethrin (Sellami et al. 2014). Oxidation of spindle fibers and microtubules in protein structure also leads to inhibition of the function of these proteins, disruption of the mitotic cycle, and MN formation. Zoex protects cells against such oxidations and ensures the continuation of the normal mitotic cycle. Phenolic compounds such as gingerol and shogaol in Zoex have the effect of reducing oxidative stress and increasing the levels of antioxidants such as GSH. This effect also neutralizes oxidative damage in cells and protects proteins such as spindle and other macromolecules from damage (Kuhad et al. 2006; Dugasani et al. 2020). Okesola et al. (2019) reported that *Z. officianale* reduced the frequency of formation of MN and improved the rates of MI, and reported that this protective property was due to active components such as phenols, saponins, and alkaloids in ginger. As a result, permethrin showed a genotoxic effect by causing a decrease in MI rates and an increase in MN frequency, while the Zoex application provided protection against cytotoxicity, and this protection was thought to be related to the active compounds in Zoex.

**Table 2:**
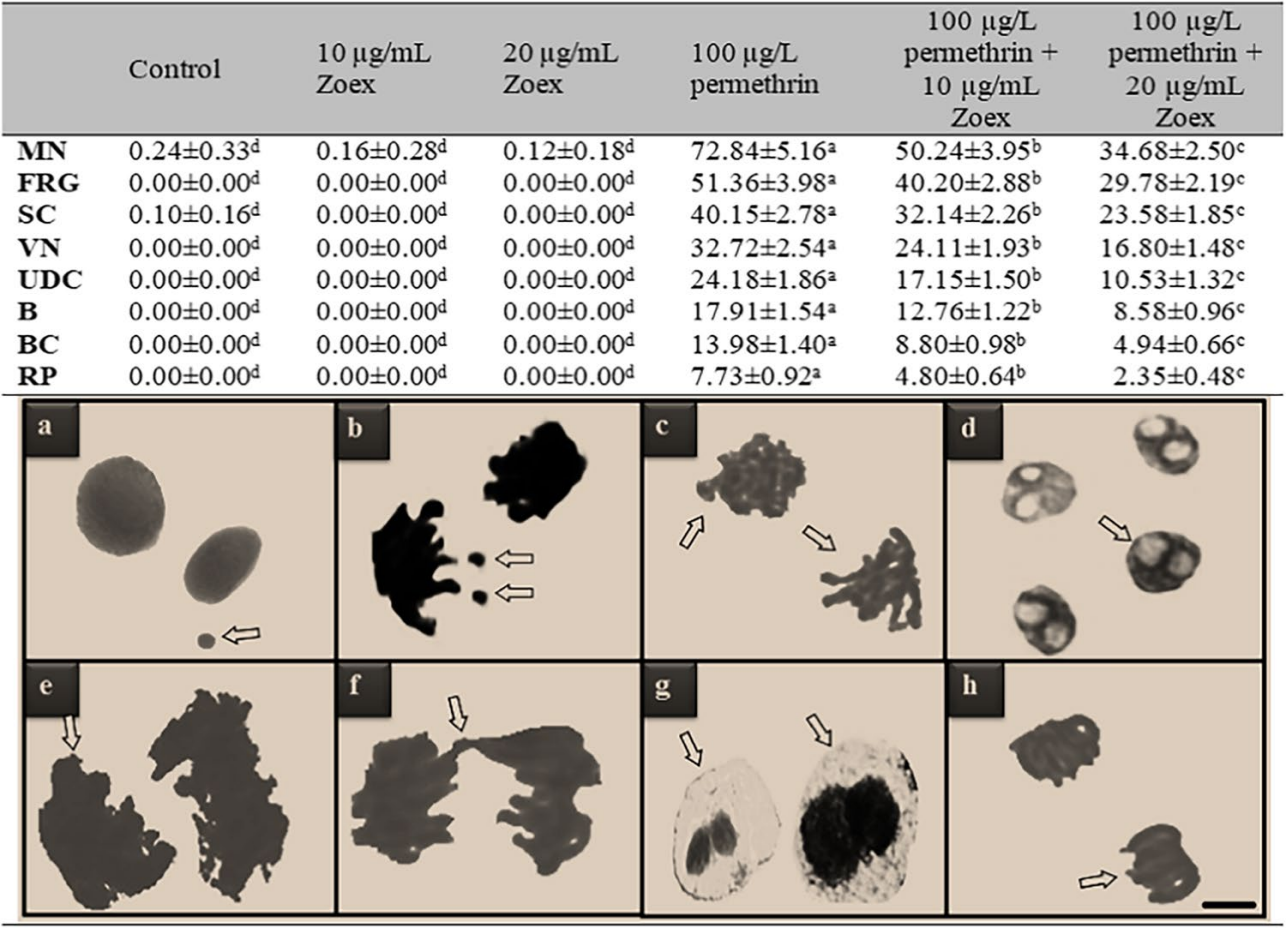
Protective role of Zoex against permethrin-induced genotoxicity

*MN* micronucleus (a), *FRG* fragment (b), *SC* sticky chromosome (c), *VN* vacuolated nucleus (d), *UDC* unequal distribution of chromatin (e), *B* bridge (f), *BC* binuclear cell (g), *RP* reverse polarization (h). Values shown with different letters in the same column are statistically significant. Bar: 10 µm

### Genotoxic effects

**Fig. 4:**
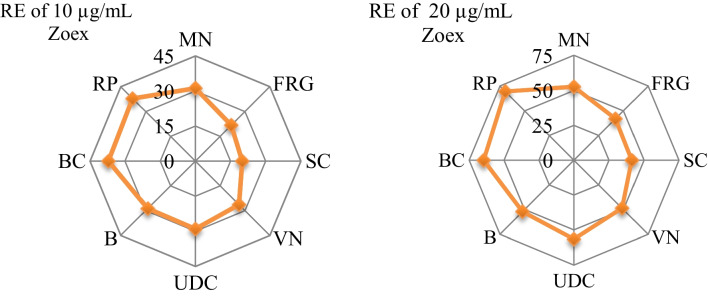
Recovery effects (RE) of the Zoex application against MN and chromosomal aberrations

A chromosomal aberration assay was performed to evaluate the possible genotoxic effects of permethrin and Zoex applications on *A. cepa* root tip cells and the results are given in Table 2. While no chromosomal aberrations were found in only Zoex applied groups, statistically insignificant sticky chromosome formation was found in the control group (*p* > 0.05). High rates of chromosomal aberrations formations were detected in group IV, which was administered only permethrin. While fragments occur with the highest frequency among chromosomal aberrations, sticky chromosomes, vacuolated nucleus, unequal distribution of chromatin, bridge, binuclear cells, and reverse polarization are the other chromosomal aberrations. The fact that permethrin causes fragment at a high rate shows that it induces breaks in DNA. In the later stages of cell division, these fragments become MN. Another aberration induced by permethrin is known as the sticky chromosome, which develops as a result of increased depolymerization of DNA, chromosomal condensation, and partial dissolution of nucleoproteins. Sticky chromosomes are a sign of extremely harmful consequences because they are frequently irreversible and may cause cell death. Vagrant chromosomes, which are detected with high frequency as a result of permethrin application, act separately from the chromosome group that is pulled to the poles, causing unequal separation of the chromosome number in daughter cells (Khanna and Sharma 2013). The fact that permethrin induces different types of chromosomal aberrations and that each abnormality occurs with different mechanisms indicates that permethrin does not exhibit a specific genotoxic effect but triggers chromosomal aberration formations with multiple mechanisms. Falcioni et al. (2010) reported that 150 mg/kg permethrin caused DNA-DNA cross-links and DNA damage due to oxidative stress in rats. The Zoex application reduced the genotoxic effects of permethrin. A total of 10 µg/mL Zoex regressed the chromosomal aberrations induced by permethrin in the range of 20.1–37.9%, while 20 µg/mL Zoex decreased the frequency between 41.3 and 69.5% (Fig. 4). The protective effects of Zoex against the chromosomal aberrations can be explained by its antioxidant activity. Permethrin induces chromosomal aberrations by causing DNA oxidation. Zoex prevents DNA damage by reducing the oxidative stress induced by permethrin and provides protection against chromosomal aberrations. Zoex is thought to have an antioxidant effect that protects against free radicals and thus may reduce genotoxic effects. This effect of Zoex is closely related to its active ingredients, and the gingerol, zingerone, and shogaol it contains are responsible for most of its biological effects. Shogaol is an activator of Nrf2 and therefore provides high protection against diseases caused by oxidative stress. Gingerol, detected in the Zoex content, shows a strong antioxidant effect by preventing the expression of cyclooxygenase and the production of ROS induced by exogenous sources (Kim et al. 2017). Due to the cumulative effect of all these phytochemicals, Zoex has potent antioxidant activity. Zoex, which has antioxidant activity by chelating metal ions and scavenging hydroxyl and hydrogen peroxide radicals, also has a protective effect against DNA damage. This effect is achieved by preventing DNA adducts and protecting DNA chains from free radical attack. The results obtained in our study are also confirmed by studies in the literature. Jeena et al. (2016) found that Zoex and active compounds prevented the negative effects of gamma radiation on cellular DNA. Similarly, Al-Amoudi (2018) reported that Zoex has protective properties against genotoxicity induced by lambda-cyhalothrin, a pyrethroid insecticide such as permethrin. As a result of the chromosomal abnormality test, it was determined that permethrin induced various types of abnormalities and the frequency of abnormalities decreased as a result of the Zoex application.

### Molecular docking-supported cytotoxicity and genotoxicity mechanism

**Fig. 5:**
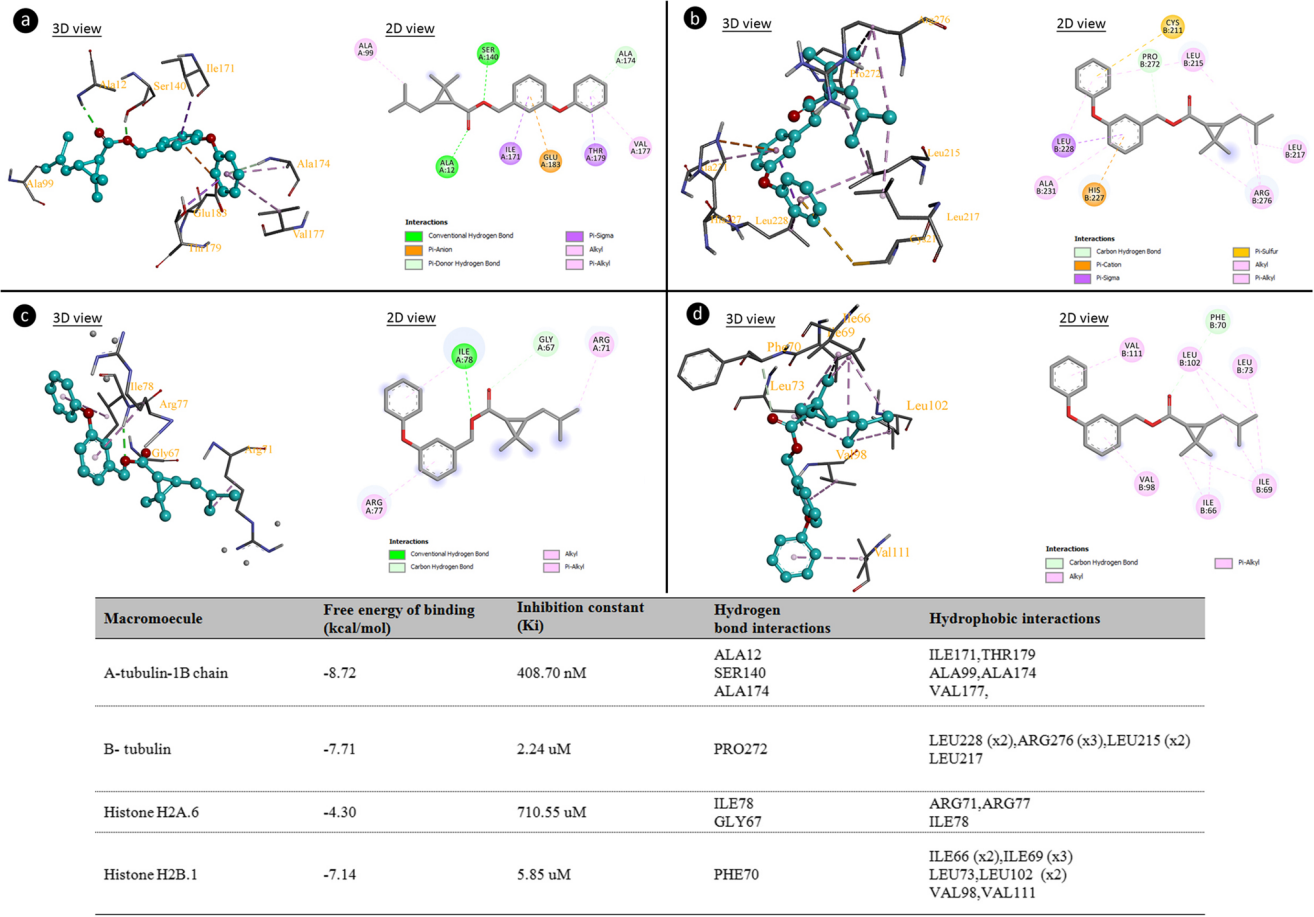
Interaction of permethrin with α- tubulin (**a**), β-tubulin (**b**), Histone H2A.6 (**c**), Histone H2B.1 (**d**)

**Fig. 6:**
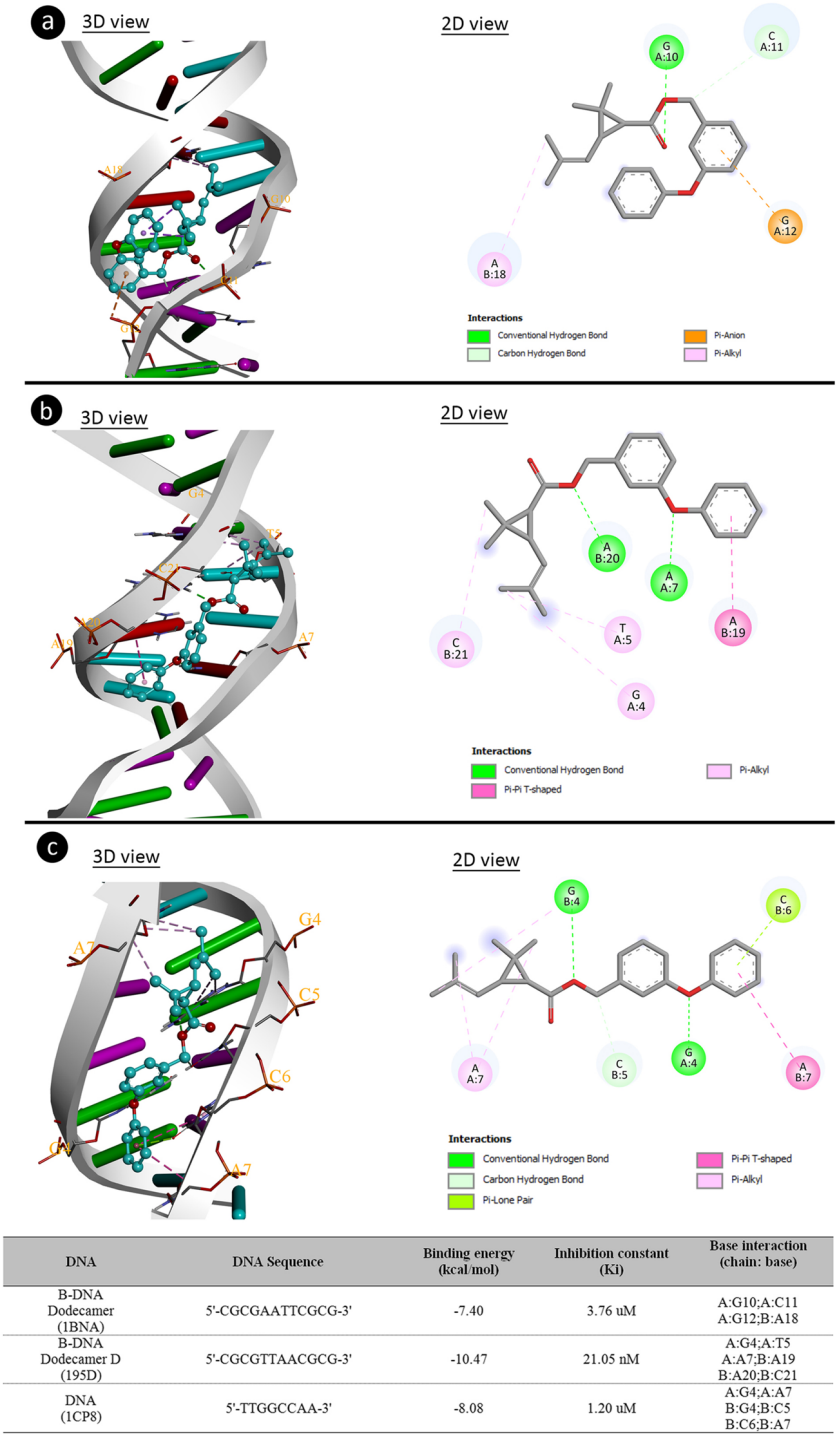
Permethrin-DNA interaction BNA (**a**), 195D (**b**), 1CP8 (**c**)

Molecular docking is a computational modeling technique used to predict and analyze molecular interactions between a small ligand molecule, such as permethrin in this study, and a target macromolecule, such as proteins (tubulin, histones) or DNA. The primary objective is to foresee the three-dimensional arrangement and the binding affinity of the complex formed by the ligand and the target. This prediction is based on the geometric complementarity and intermolecular forces between the ligand and the binding site on the macromolecule (Agu et al. 2023). The mechanisms of cytotoxicity and genotoxicity of permethrin were elucidated by molecular docking. To determine the mechanism of cytotoxic action, α- and β-tubulin proteins were chosen as target ligands. The interactions of permethrin and α/β-tubulin proteins are shown in Fig. 5. Permethrin interacts with alanine and serine amino acids in the α-tubulin via hydrogen bonds. It also forms hydrophobic interactions with amino acids such as leucine and threonine. Permethrin has a binding energy of − 8.72 kcal/mol and an inhibition constant of 408.70 nM for the α-tubulin. Similarly, permethrin interacts with the β-tubulin, with these interactions occurring through hydrogen bonding with proline and hydrophobic interactions with leucine amino acids. Mitotic spindles consist of microtubules that separate chromosomes. α- and β-tubulin proteins polymerize to form microtubules. Damage to these proteins can also lead to disruption of microtubule polymerization. The possible interaction of permethrin with tubulin proteins leads to abnormalities in the polypeptide structure. As a result of this abnormality, microtubule polymerization is prevented and chromosome movement to the poles is restricted, leading to disruption of mitotic stages and chromosome abnormalities. Permethrin-tubulin interactions are the basis for the cytotoxic impact of permethrin, which is demonstrated by a reduction in the rate of MI in *A. cepa*. The genotoxic effect of permethrin may occur as a result of permethrin-histone (Fig. 5) and permethrin-DNA interactions (Fig. 6). Permethrin has binding energies of − 4.30 and − 7.14 kcal/mol, inhibition constant of 710.55 µM and 5.85 µM with histone H2A.6 and Histone H2B.1 proteins, respectively. Permethrin and histone proteins interact hydrophobically through a variety of amino acid residues. Histones are proteins that bind to DNA and prevent it from knotting and protect it from damage. In addition, histones also play an important role in gene regulation and DNA replication. Disruption of the interaction between histone and DNA, which are bound by electrostatic interactions and hydrogen bonds, leads to impaired DNA integrity (Saha et al. 2017). Permethrin interacts with histone proteins, weakening the DNA-histone bond, leading to the degradation or dissolution of DNA and chromosome structure. One of the mechanisms for the genotoxic effect of permethrin may be permethrin-histone interactions. After molecular docking analysis with different DNA sequences, permethrin was found to interact with G10, C11, and G12 in the A chain and with A18 in the B chain of 1BNA. Permethrin interacted with G4, T5, and A7 in the A chain and A19, A20, and C21 in the B chain of 195D. The interaction of permethrin and 1CP8 resulted in a binding energy of − 8.08 kcal/mol. As a result of molecular docking with three different DNA target molecules, it was found that permethrin can affect DNA structure by binding to regions rich in G-C-G, G-T, A-A-C, and G-C-C-A bases. In addition, permethrin has an intercalation potential. Intercalating substances have a wide range of biological effects on DNA. Among these impacts include inhibition of DNA or RNA synthesis, frameshift mutations, and protein-associated DNA breaks. Permethrin was found to have a genotoxic effect both by binding to different DNA and by functioning as an intercalator agent. Compounds that are able to insert into DNA lead to an increase in clastogenic effects. As a result of these effects, abnormalities in DNA synthesis, strand breaks, insertion, deletion, or rearrangement of chromosomes may occur (Ferguson and Denny 2007).

### DNA-permethrin interaction confirmed by spectral shift

**Fig. 7:**
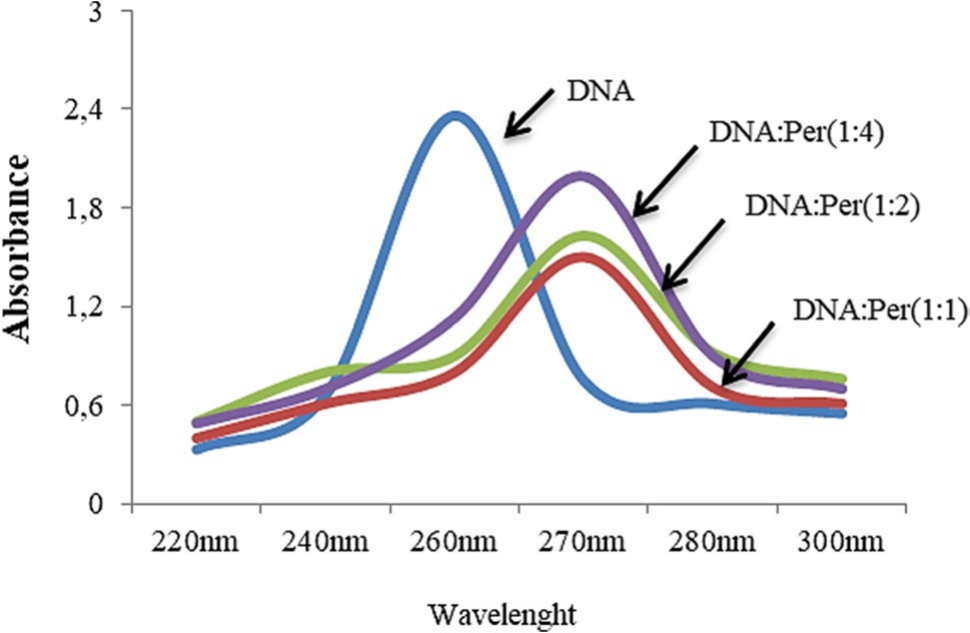
UV-Vis spectrum of *Allium* DNA in the presence and absence of permethrin

As shown in molecular docking, the mechanism of permethrin genotoxicity in this work can be linked to DNA-permethrin interaction. In order to verify this connection, increasing concentrations of permethrin solution were added to DNA extracted from *Allium* root tips, and the alterations in the UV absorption spectrum were measured (Fig. 7). *Allium* DNA isolated in this study showed a characteristic maximum peak at 260 nm. The interaction of permethrin with DNA caused bathochromic and hypochromic shifts in the UV spectrum. The bathochromic shift refers to the increase in wavelength, and a shift from 260 to about 270 nm was observed in the DNA spectrum after DNA-permethrin interaction. The hypochromic shift refers to the decrease in the absorption of a substance, and the absorbance of the DNA solution decreased after the DNA-permethrin interaction. DNA with an absorbance of 2.36 at 260 nm showed an absorbance of 1.99 at 270 nm when mixed with permethrin at 1:4 ratios. According to Ozluer and Satana Kara (2014), the hypochromic change shows that a molecule engages DNA through its intercalation-binding mechanism. The intercalation property of permethrin is indicated by the decrease in the absorbance of the DNA: permethrin solution compared to the DNA absorbance. Molecular docking was used to establish this feature of permethrin, and spectrum analysis verified it. Without forming any covalent bonds, a molecule is stacked between DNA base pairs during intercalation. The majority of DNA intercalators are promutagenic. Additionally, intercalation results in DNA breaks, unravels supercoiled DNA, and messes up the way that DNA interacts with regulatory and binding proteins (Ferguson and Denny 2007; Guengerich 2014). The increased frequency of chromosomal abnormalities and MN observed in this study may be caused by DNA-permethrin interaction, which also explains the mechanism of genotoxicity.

### Comet assay

**Fig. 8:**
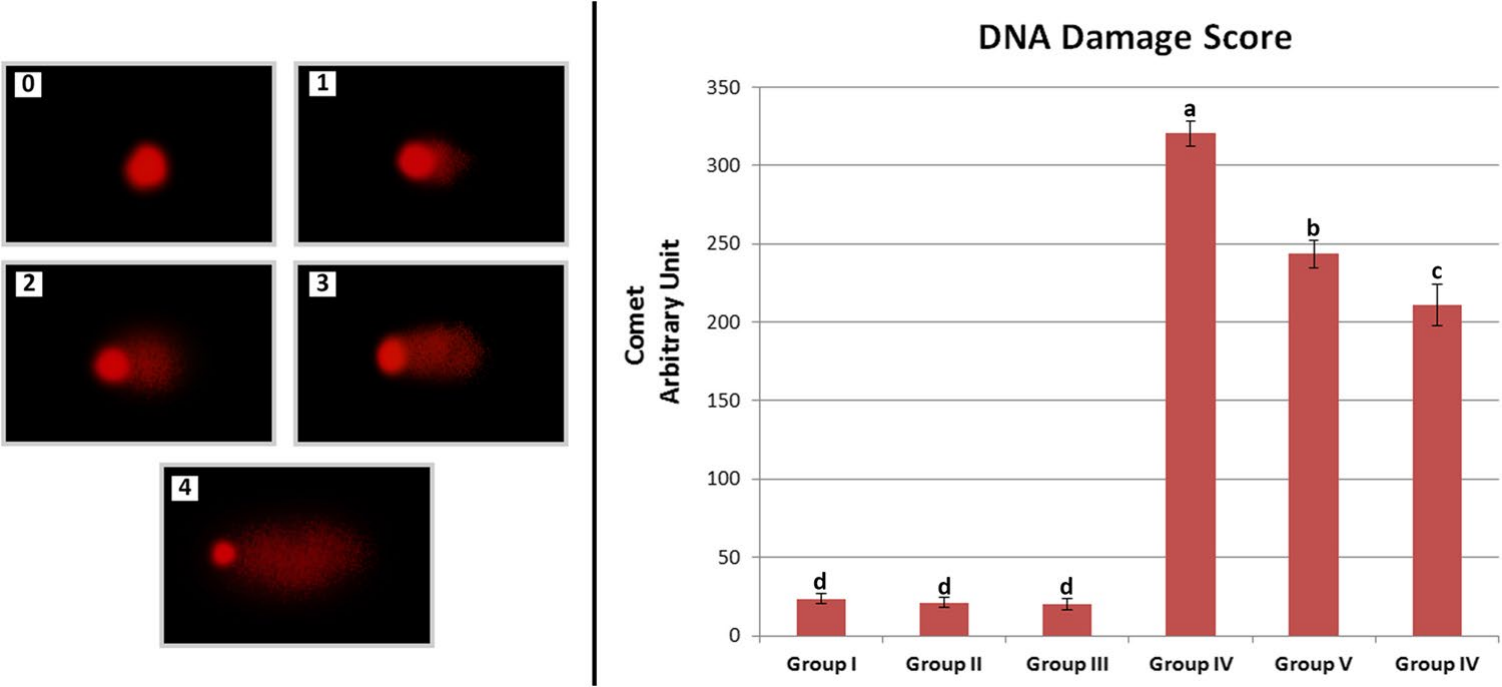
The effect of permethrin and Zoex application on DNA of *A. cepa* (0, no damage; 1, low damage; 2, moderate damage; 3, high damage; 4, extreme damage

The effects of permethrin and Zoex administration on DNA fragmentation are summarized in Fig. 8. Zoex administration alone did not cause DNA damage and there was no statistically significant difference (*p* > 0.05) between the control group (group I) and the groups treated with Zoex alone (groups II and III). While the average DNA damage value in the control group was 23.33 ± 7.97, a sharp increase occurred in the group IV, which was administered 100 µg/mL permethrin, and the average DNA damage value was 320.50 ± 7.83. Zoex application with permethrin showed a protective effect depending on the applied dose and the DNA damage score was calculated as 243.50 ± 8.67 in group V administered 100 µg/mL permethrin + 10 µg/mL Zoex and 210.67 ± 13.07 in group VI administered 100 µg/mL permethrin + 20 µg/mL Zoex. The data obtained showed that permethrin application caused DNA damage, and Zoex application showed a protective effect depending on the dose. Differences in DNA damage scores between groups I–III and groups IV–VI are statistically significant (*p* < 0.05).

### Anatomical alterations

**Fig. 9:**
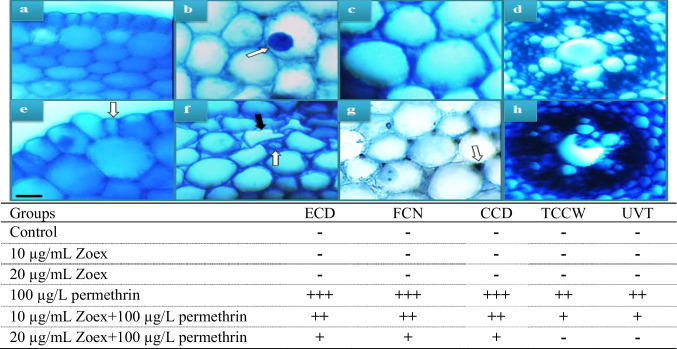
Meristematic cell damage caused by permethrin. Epidermis cells of control (**a**), cell nucleus (*oval) in control* (**b**), cortex cells in control (**c**), vascular tissue in control (**d**), epidermis cell damage (**e**), flattened cell nucleus-*white arrow*, cortex cell damage-*black arrow* (**f**), cortex cell wall thickening (**g**), and unclear vascular tissue (**h**). ECD, epidermis cell damage; CCD, cortex cell damage; TCCW, thickening of cortex cell walls; UVT, unclear vascular tissue; FCN, flattened cell nucleus. (-) no damage, ( +) minor damage, (+ +) medium damage, (+ + +) severe damage. Bar 10 µm

The anatomical damages induced by permethrin and Zoex in the meristematic cells and the frequency of damage in each group are shown in Fig. 9. While no abnormalities were observed in meristematic tissues in the control group and in the group to which only Zoex was administered, the abnormalities shown in Fig. 9 occurred in the group to which permethrin was administered. These abnormalities are related to permethrin toxicity in the cells or to the tolerance mechanisms of the cells to permethrin. To prevent permethrin from entering the cell, chemicals such as lignin, cellulose, suberin, and cutin are accumulated in the cell wall, and the wall becomes thickened. According to Shu et al. (2012) and Singh et al. (2015), this change acts as a barrier against harmful chemicals and prevents toxic compounds from entering the vascular tissue. Nuclear shape change is another abnormality, and genotoxic and biochemical changes induced by permethrin may be the cause of nuclear flattening. Changes in nuclear shape may result from deterioration of nuclear volume, protein concentration, DNA integrity, and density (Dahl et al. 2008; Dauer and Worman 2009). Similarly, Yalçın et al. (2019) found that numerous morphological changes, such as deformation of epidermal cells and thickening of cell walls, were observed in root anatomy under chemical-induced stress. Other abnormalities caused by permethrin include disorganized vascular tissue and cellular damage to the epidermis. The cause of all these abnormalities could be permethrin-induced oxidative stress, cytotoxic, and genotoxic effects. Such anatomical abnormalities prevent the plant from taking up nutrients and transporting them to other tissues and may also retard growth. Zoex application with permethrin resulted in a decrease in the incidence of anatomical damage. The application of 10 µg/mL and 20 µg/mL Zoex provided significant protection by causing a significant decrease in anatomical damage, with the most significant protection obtained in the group VI where 20 µg/mL Zoex was applied. In the group VI, no thickening of cortex cell walls and no unclear vascular tissue were observed, while damage to epidermal cells, flattening of the nucleus, and damage to cortex cells were significantly reduced. The protective effect of Zoex on the anatomical structure may be related to the reduction of physiological, biochemical, and cytogenetic abnormalities induced by permethrin in meristem cells in the presence of Zoex.

## Conclusion

There are numerous studies on the toxicity of permethrin, a synthetic pyrethroid insecticide, in various non-target organisms, but there are not enough studies examining toxicity in plants. This study is the first to show the toxicity of permethrin in *Allium cepa*, a bioindicator plant, and the protective effect of Zoex against this toxicity. In vivo studies examining the toxic effects of chemicals, the protective effects of natural products against these effects, and the underlying mechanisms are very valuable. In this study, permethrin-induced oxidative stress by increasing MDA levels and decreasing GSH levels, and showed genotoxic effects by inducing MN and chromosomal abnormalities. These toxic effects were also reflected in the regression of germination and anatomical changes. In silico interaction of permethin with DNA sequences, tubulin and histone proteins confirmed its genotoxic and cytotoxic effects. Molecular docking and spectral shift analysis revealed that permethrin acts as an intercalating agent and interact with DNA non-covalently. Thus, the genotoxicity mechanism of permethrin can be explained by the intercalation function and the disruption of DNA integrity. The effects of permethrin on *Allium cepa*, a non-target organism, may also provide a preliminary prediction for eukaryotic organisms. However, detoxification systems, especially in mammals, may alter the extent of permethrin toxicity. Therefore, toxic effects on all non-target organisms should be investigated and dose ranges with lower toxicity identified. Natural products containing such potent antioxidant agents are very important in eliminating the toxic effects of chemicals. Zoex provided dose-dependent protection against permethrin toxicity, and this protection was attributed to the active ingredients it contained. This study will guide many studies that elucidate the mechanisms by combining data obtained from in vivo studies with bioinformatics data.

## Data Availability

All data generated or analyzed during this study are included in this article.
